# *Optix* and *cortex/ivory/mir-193* again: the repeated use of two mimicry hotspot loci

**DOI:** 10.1098/rspb.2024.0627

**Published:** 2024-07-24

**Authors:** Anna Orteu, Emily A. Hornett, Louise A. Reynolds, Darrell J. Kemp, Gabriele Gloder, Ian A. Warren, Gregory D. D. Hurst, Simon H. Martin, Chris D. Jiggins

**Affiliations:** ^1^ Tree of Life Programme, Wellcome Sanger Institute, Hinxton, UK; ^2^ Department of Zoology, University of Cambridge, Cambridge, UK; ^3^ Institute of Infection, Veterinary and Ecological Science, University of Liverpool, Liverpool, UK; ^4^ Vector Biology, Liverpool School of Tropical Medicine, Liverpool, UK; ^5^ Department of Biology, University of Oxford, Oxford, UK; ^6^ School of Natural Sciences, Macquarie University, Sydney, New South Wales 2109, Australia; ^7^ Department M2S, KU Leuven, Willem De Croylaan 46, Leuven B-3001, Belgium; ^8^ Institute of Evolutionary Biology, University of Edinburgh, Edinburgh, UK

**Keywords:** cortex, ivory, mimicry, convergent evolution

## Abstract

The extent to which evolution is repeatable has been a debated topic among evolutionary biologists. Although rewinding the tape of life perhaps would not lead to the same outcome every time, repeated evolution of analogous genes for similar functions has been extensively reported. Wing phenotypes of butterflies and moths have provided a wealth of examples of gene re-use, with certain ‘hotspot loci’ controlling wing patterns across diverse taxa. Here, we present an example of convergent evolution in the molecular genetic basis of Batesian wing mimicry in two *Hypolimnas* butterfly species. We show that mimicry is controlled by variation near *cortex/ivory/mir-193*, a known butterfly hotspot locus. By dissecting the genetic architecture of mimicry in *Hypolimnas misippus* and *Hypolimnas bolina*, we present evidence that distinct non-coding regions control the development of white pattern elements in the forewing and hindwing of the two species, suggesting independent evolution, and that no structural variation is found at the locus. Finally, we also show that orange coloration in *H. bolina* is associated with *optix,* a well-known patterning gene. Overall, our study once again implicates variation near the hotspot loci *cortex/ivory/mir-193* and *optix* in butterfly wing mimicry and thereby highlights the repeatability of adaptive evolution.

## 1. Introduction

Convergent evolution, defined as the independent evolution of similar traits in different lineages, often in response to similar environmental pressures, has long fascinated evolutionary biologists [1]. More recently, genetic analysis has revealed that convergent phenotypes fairly commonly involve similar genetic changes [2,3]. Many examples of this come from coloration phenotypes, where certain ‘hotspot genes’ or homologous loci have been repeatedly linked to both similar and divergent phenotypes. These genetic hotspots are hypothesized to represent loci that maximize changes to the trait while minimizing pleiotropic effects [1]. However, the apparent repeatability of evolution can be the result of different mechanisms that might affect the likelihood of those events. The evolution of parallel changes can originate from independent mutations at the same gene or locus (for example, [4]) and might represent a somewhat rare event. Alternatively, introgression of adapted alleles from other lineages (for example, [5]) and selection on shared ancestral variation present at the locus after lineage divergence (i.e. standing genetic variation; for example, [6]) can also lead to repeated evolution without relying on independent mutational events happening in distinct lineages [1,7].

Butterfly wing phenotypes are a well-studied system for understanding the evolution of adaptive traits and the genetic basis of convergent phenotypes [8–11]. Much of the attention has focused on mimicry in tropical butterflies such as *Heliconius* and *Papilio* species. *Heliconius* is a genus of tropical butterflies with striking Müllerian mimicry, in which multiple sympatric and unpalatable species evolve to resemble one other, thereby sharing the costs of teaching predators [12]. Four major effect genes have been associated with wing convergent phenotypes in several *Heliconius* species: *cortex/ivory/mir-193, aristaless1, WntA* and *optix* [4,13–15]. Mimicry between closely related *Heliconius* species is usually associated with the sharing of allelic variants at these loci, whereas mimicry between distant *Heliconius* species usually involves de novo mutations at the same loci. Interestingly, *cortex* had been thought to control scale morphology and the switch between white/yellow and black/red in *Heliconius* [16] and to also be implicated in colour phenotypes in other divergent Lepidoptera species [4,17–22]. However, new evidence has shown that those phenotypes are controlled by the long non-coding RNA *ivory*, which overlaps with *cortex’s* coding sequence [23,24], and the nearby microRNA *mir-193* [25]. Hereafter, we refer to this region as the *cortex/ivory/mir-193* locus for simplicity. Similarly to *cortex/ivory/mir-193*, *WntA* and *optix* have been shown to be involved in wing patterning in several other Lepidoptera highlighting the repeatability of the genetic control of wing phenotypes in butterflies and moths [14,15,26,27]. However, the extent of the re-use of similar genes for wing phenotypes is still largely unexplored. Dissecting the genetic architecture of adaptive wing phenotypes in other species will improve our understanding of the evolution of adaptive alleles.

**Figure 1.**
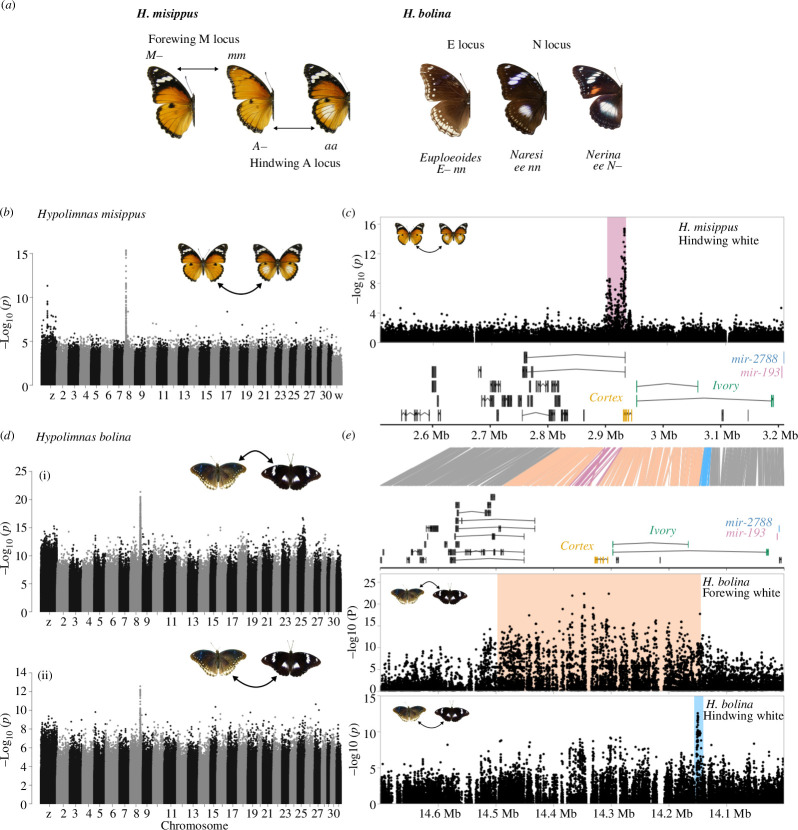
Variation near *cortex/ivory/mir-193* is associated with white coloration in *H. misippus* and *H. bolina*. (*a*) Wing pattern in *H. misippus* is controlled by the biallelic, Mendilian M locus (forewing) and the A locus (hindwing) with two alleles and intermediate inheritance. In *H. bolina,* two loci, E and N, control the presence and absence of white and orange spots in the wings, respectively. (*b–d*) GWAS for the presence/absence of a hindwing white spot in *H. misippus* (*b*) and forewing subapical band (*d*(i)) and a hindwing white spot (*d*(ii)) in *H. bolina* reveal association peaks on chromosome 8. (*c*) Zooming into the associated region in *H. misippus* shows that the *cortex, ivory* and *mir-193* are just downstream of the association peak. (*c*) Similarly, zooming into the two association peaks for hindwing and forewing white in *H. bolina* shows that *cortex, ivory* and *mir-193* are found by the association peak.

Two species of *Hypolimnas* butterflies, *Hypolimnas misippus* and *Hypolimnas bolina*, show female-limited polymorphic Batesian mimicry [28–30]. In Southeast Asia, *H. bolina* is a recognized Batesian mimic of multiple *Euploea* species, while in the South Pacific Islands and Australia, it has several non-mimetic forms. The genetic basis of this wing pattern variation was extensively studied by Clarke & Sheppard [29], who hypothesized that three of the main wing morphs, *nerina*, *naresi* and *euploeoides*, are determined by two autosomal loci. They hypothesized that the E locus controls the differences between the all-brown mimetic morph *euploeoides* and the *naresi* morph, which presents a hindwing white spot and a subapical white band in the forewing (figure 1). While the N locus controls the presence of forewing orange patch seen in *nerina* wings.

Polymorphic *H. misippus* females show a detailed resemblance to the four morphs of the unpalatable *Danaus chrysippus*. Genetic variation in *D. chrysippus* has been mapped to three loci, offering an opportunity to compare the genetic basis for wing pattern variation on both a model and its Batesian mimic species. In *H. misippus,* forewing and hindwing phenotypes are controlled by independent loci, and the matching mimetic morphs are achieved by different combinations of alleles of the forewing and hindwing forms (figure 1) [18,28,30–32]. The M locus controls the differences in forewing phenotype, with the dominant allele *M* producing the black-and-white *misippus* wings, while the recessive *m* allele produces all-orange wings and intermediates known as *inaria* and *immima* [18,33]. Interestingly, the M locus has been narrowed down to a non-coding region around which no known Lepidoptera colour genes are found (refer [18,33]). This shows that wing colour phenotypes can be controlled by novel genes not described in other species.

Hindwing colour in *H. misippus* varies continuously from orange to white and is controlled by the A locus, whose dominant allele produces hindwing white and presents incomplete dominance [32]. A suppressor locus, the S locus, has been hypothesized to counteract the effect of the A locus by limiting the presence of white in the hindwing. Furthermore, the A locus has been hypothesized to be epistatic with the M locus, controlling the switch between *inaria* and *immima* forewing phenotypes. In summary, both *H. misippus* and *H. bolina* have a simple genetic basis for polymorphic female-limited Batesian mimicry. Crucially, in both species, white coloration in the hindwing is continuous but controlled by one or two loci of major effect. Separated by 8 million years (Myr) of evolution [34], *H. misippus* and *H. bolina* are a good case study to explore the genetics of wing mimicry and the extent of gene re-use in the evolution of these phenotypes.

Here, we investigate the genetic basis of Batesian mimicry in *H. misippus* and *H. bolina*. We use whole-genome analysis of linked-read and short-read sequencing data, synteny and phylogeny to investigate the genetic control and evolution of hindwing and forewing white coloration in the genus and compare it to other Lepidoptera.

## 2. Material and methods

### (a) Sample collection, processing and analysis of *H. misippus*

To explore the genetic basis of hindwing coloration in *H. misippus*, samples described by Orteu *et al*. [33] were used (electronic supplementary material, table S4). Briefly, 335 individuals were collected in different parts of Africa and preserved in 100% ethanol or sun dried. DNA was then extracted from the samples and libraries prepared using custom protocols (described in Orteu *et al*. [33]) and sequenced using haplotagging, a linked-read sequencing technique. BX tags including barcode information of the linked reads were included in the read information field. Low-quality ends and adapters were trimmed using TRIMMOMATIC [35]. Trimmed and filtered reads were mapped to the *H. misippus* reference genome (*HypMisi_v2,* [36]) using BWA-MEM [37], and PCR duplicates were marked using Picard tools (broadinstitute.github.io/picard). SNPs were called using bcftools v. 1.11 [38], imputed using STITCH [39] and phased using HapCut2 [40].

### (b) Collection and processing of wild *H. bolina* samples

In total, 214 wild *H. bolina* were collected from the island of Rurutu in French Polynesia in 2004, 2005, 2007 and 2013, Moorea in 2005 and 2010 and from Cairns, North Queensland, Australia, in 2018, or purchased from a butterfly farm (originating from Southeast Asia; electronic supplementary material, table S3). First, DNA was extracted following a custom protocol that uses PureLink buffers and homemade magnetic beads. To do that, a small piece of thorax tissue (1/10) was dissected and placed in an eight-tube PCR strip, to which 45 µl of PureLink Digestion buffer and 10 µl of proteinase K (20 mg/ml) were added and the samples incubated for 2–3 h at 58°C with shaking (500 r.p.m.), manually inverting them vigorously every 30 min. After that, 2 µl of RNAse (DNAse free) was added to each sample, mixed by inversion and incubated for 10 min at room temperature. Tubes were then centrifuged briefly, and 45 µl of PureLink Lysis Buffer was added before mixing and incubating the samples for 30 min at 58°C with shaking (500 r.p.m.). Afterwards, to pellet any undigested solids, the samples were centrifuged at 4000*g* for 10 min at room temperature. Following that, the DNA was extracted from the lysate using a homemade magnetic bead mix. First, 37.5 µl of magnetic bead mix was added in each well of a 96-well plate. Then, 75 µl of lysate was transferred to the well plate and mixed by pipetting. Two rounds of 80% ethanol clean-ups were then performed, placing the well plate in a magnetic stand. After the second round of clean-up, 50 µl of 10 mM Tris at pH 8 was added to elute the DNA. The mix was incubated for 15 min at 45°C, and then the samples were mixed and incubated for 20 min at room temperature. Finally, the samples were placed on the magnet stand, and the clean DNA was transferred to a fresh strip tube.

From the extracted DNA, libraries were prepared following a method based on Nextera DNA Library Prep (Illumina, Inc.) with purified Tn5 transposase [41]. PCR extension with the N701-N800 i7-index primer and the N501-N508 and N5017 i5-index primers was performed to barcode the samples. Library purification and size selection were done using the same homemade beads as mentioned above. Pooled libraries were sequenced to approximately 7X coverage by Novogene Cambridge, UK.

Reads were first trimmed using fastp [42], which performs quality control and trims low-quality ends and adapters. Then, processed reads were mapped to the two reference genomes, *HypMisi_v2* and *HypBol_v1,* produced in Orteu *et al*. [36] using BWA-MEM2 [43], and PCR duplicates were marked using the MarkDuplicatesSpark from GATK [44]. SNPs were called on each chromosome separately using bcftools v. 1.11 [38] mpileup requesting the INFO/AD,AD,DP,DV,DPR,INFO/DPR,DP4,SP tags to output (-a option), setting the minimum mapping quality to 10 (-q) and the minimum base quality to 20 (-Q), ignoring Read Group tags (--ignore-RG) and removing duplicates (-F 1024). The output was piped from bcftools mpileup directly to bcftools call to obtain the final vcf files of called SNPs using the alternative model for multi-allelic and rare-variant calling (--multiallelic-caller), including only variants in the output (--variants-only) and the fields GQ and GP (-f GQ,GP). Thereafter, the data were filtered based on genotype quality (>30) and depth (>2 and<12). Thresholds were set after exploring a subset of the data.

### (c) Sample phenotyping

Once samples had been collected, the forewings and hindwings of *H. misippus* individuals were photographed in a standardized set-up consisting of a CS-920S Copy Stand holding a Cannon EOS 700D camera with a Cannon EFS 60 mm macro lens 43 cm above the wings. Two Godox SK400 lights were used, and the wings were placed in a green background with a white (Ocean Optics, Inc. WS-1) and a grey balance checker (Grey White Balance Colour Cards). Phenotypes of *H. misippus* were then scored from the photographs following Gordon & Smith [32], in which hindwings are classified according to the number of sections of the wing (interveins) containing white scales. In this scale, 8 is the maximum amount of white possible and 0 is the minimum, with the wing being fully orange. For the categorical classification, 0 was assigned to any individual with a 0 in the continuous scale and 1 to all the other scores (1–8). *Hypolimnas bolina* samples were phenotyped by the presence (1) or absence (0) of white patches in the forewing and hindwing separately. Also, forewings were also scored by the presence (1) or absence (0) of an orange patch. All phenotypes are included in electronic supplementary material, tables S1–S3.

### (d) Analysis of *H. bolina*-reared individuals and quantitative trait locus (QTL) mapping

To identify the genetic basis of wing mimicry in *H. bolina,* a QTL mapping analysis was performed using family samples from Orteu *et al*. [36] (electronic supplementary material, table S2). Briefly, two families (Family 1 and Family 2) were reared as follows. Female *H. bolina* purchased from Stratford Butterfly Farm (originating from the Philippines) were mated to wild-caught males from Mo’orea (French Polynesia) in French Polynesia (Gump research facility). Female Philippines/Mo’orea F1 hybrids were mated to pure Mo’orea F1 males. The F2 offspring of one of these crosses is family Family 1 (so they are Philippines/Mo’orea/Mo’orea). Male Philippines/Mo’orea F1 hybrids were mated to pure Philippine F1 females. The F2 offspring of one of these crosses is family Family 2 (so Philippines/Mo’orea/Philippines). DNA was extracted, and libraries were prepared using the same custom protocols as for the wild individuals. Samples were then sequenced to approximately 11× for the offspring and approximately 20× for the parents. Low-quality ends and adapters were trimmed using TrimGalore! [45], reads were mapped using BWA-MEM [37] and PCR duplicates were marked with Picard tools. SNPs were called using bcftools v. 1.11 [38], and QTL mapping was performed using the R package qtl2 [46].

### (e) Genome-wide association analysis in *H. bolina*

To confirm and further explore the results of the QTL mapping, 45 wild Samoan *H. bolina* from Hornett *et al*. [47] were used for a preliminary association analysis, together with the reared individuals (electronic supplementary material, table S2). GWAS was performed using GEMMA (REF), correcting for population structure and relatedness. First, a principal component analysis (PCA) was generated using Plink v. 1.9 [48]. Then the relatedness matrix was built with GEMMA [49] and used, together with the first 20 PCs, as input for the linear mixed-model utility of GEMMA. This approach has been previously used with good results [18].

The results from QTL mapping and the preliminary GWAS produced a broad associated region. To narrow it down, the 214 wild *H. bolina* samples described above were used for a second GWAS analysis. To do that, a PCA analysis in *H. bolina* and *H. misippus* was first carried out using Plink v. 1.9 [48] (electronic supplementary material, figure S3) and the first five principal components used as covariates in the association test, which was performed with Plink using the –assoc option. The −log_10_ significance levels were calculated using Bonferroni correction to account for multiple testing, which were 8.75 and 9.11 in *H. bolina* mapped to the *H. bolina* reference and to the *H. misippus* reference, respectively, and 8.96 in *H. misippus*, calculated from the 27 889 110, 64 636 142 and 46 088 305 SNPs used.

### (f) Chromosome naming

To facilitate the presentation of results, chromosomes for *H. misippus* and *H. bolina* have been named based on homology with *Melitaea cinxia* (unless otherwise specified). Briefly, BUSCO [50] matches using the odb_insecta10 gene set are used to infer homology. The homology between *H. misippus* and *H. bolina* chromosomes with *M. cinxia* is summarized in electronic supplementary material, table S1.

### (g) Reference genome alignments

To investigate the origin of the adapted alleles, homology between regions associated with wing phenotype in each species was explored. To identify putative orthologous regions between the reference genomes of *H. bolina* and *H misippus*, the two references, *HypMisi_v2* and *HypBol_v1,* were aligned with Satsuma2 [51] using default parameters. The resulting alignments were visualized using the asynt R functions [52].

### (h) *Ivory, mir-193* and *mir2788* annotation and gene orthology

The *ivory, mir-193* and *mir2788* nucleotide sequences from Fandino *et al*. [23,24] were used to search (BLASTn [53] with *E*-value > 1 × 10^−10^) for orthologous annotated genes in the *HypBol_v1 and HypMisi_v2* genomes, but the results did not overlap with any annotated genes. To annotate them, we used the BLASTn results. Similarly, BLASTp (*E*-value > 1 × 10^−10^) to the *H. melpomene* (Hmel2.5) and *D. melanogaster* (GCF_000001215.4_Release_6) genomes was used to find orthologous genes to the annotated genes in the region, retaining only the best hit per gene.

### (i) Read-depth analysis and identification of indels

Identification of large indels putatively associated with wing phenotype was performed by calculating read depth from the bam files using the depth utility from Samtools [38] with the -a option to output depth for all sites, including those with no reads mapping to them. The output was visualized in R using the ggplot2 package.

Individual BAM files with marked duplicates were subset for the region of interest using Tabix and merged using Samtools merge. These merged BAM files were then visualized using IGV. The candidate indels were identified through visual inspection. SNPs associated with the indel were used as proxies to identify the individuals carrying the deletion and insertion.

### (j) Phylogenetic trees

To explore the evolution of the alleles, phylogenetic trees were generated in genomic windows. To do that, genotype files were first produced using the parseVCF.py from the genomics_general toolkit (https://github.com/simonhmartin/genomics_general) using the phased vcf files produced by HAPCUT2 as input. Then neighbour-joining trees of the cortex locus were generated in windows of 50 SNPs using the BIONJ algorithm implemented in Phyml (REF), via the wrapper script phyml_sliding_windows.py of the genomics_general toolkit, setting the options –windType sites –model GTR –optimise n.

## 3. Results

### (a) Forewing white and hindwing white are controlled by independent functional elements at the E locus in *H. bolina*

Clarke & Sheppard [29] hypothesized that the differences between the all-brown *euploeoides* morph and the white-spotted *naresi* morph of *H. bolina* were controlled by a single locus of major effect, the E locus (figure 1*a*). That is, a single locus controlled the presence/absence of white patches in the forewing *and* hindwing at the same time. This locus presents a dominant *E* allele that produces the all-brown *euploeoides* wings and a recessive *e* allele that produces the white-spotted *naresi* (figure 1*a*). A single cross described here provides evidence for recombination between forewing and hindwing elements within this major locus. Given that polymorphism is female-limited, genotypic information from crosses comes only from female phenotypes. A first cross was performed between a female presenting a forewing white band and a large hindwing white patch, which would be considered a *naresi* morph. Then, a female offspring of this cross with a forewing white band, but only a reduced hindwing white patch, was mated to a wild male of uninformative phenotype as the trait of interest is female limited (Family 1, electronic supplementary material, table S2). All the female offspring of this cross had a forewing white band, while they varied in the presence and size of the hindwing patch, with 34 individuals having all-brown wings and 18 individuals with a white patch (varying in size). The segregation of hindwing but not forewing white in this cross suggests that there are two functionally distinct linked elements and that a single recombination event occurred. Tight physical linkage between the two elements would explain not only the joint inheritance of the traits but also the existence of recombinants.

### (b) GWAS for white coloration points at *cortex/ivory/mir-193* as the main candidates

Next, we identified the region of the genome controlling the presence of white elements in *H. bolina* using two datasets, that is, the E locus. Using the reared families from Orteu *et al*. [36], we performed a QTL mapping analysis, which showed that the locus associated with hindwing white variation is found in chromosome 8 (electronic supplementary material, figure S1 and table S2). In *H. bolina,* males are uninformative for the E locus genotype, as they are monomorphic. This together with the fact that there is substantial continuous variation in hindwing white hinders the correct genotyping of samples from phenotype information. Given the segregation pattern of hindwing phenotype (1:3), we deduced that one of the parents was heterozygous for the E locus and the other homozygous for the recessive allele that produces white patches (*ee*). We performed two QTL analyses assuming heterozygosity of either parent, which both identified an association peak at chromosome 8. Given that there is no recombination in female butterflies [54], when the mothers of the crosses are assumed to be heterozygous, the association can only be narrowed down to the chromosomal level (electronic supplementary material, figure S1).

To confirm this result, we used these sequenced families together with a dataset of 45 sequenced wild individuals from Hornett *et al.* [47] and performed a GWAS correcting for population structure and relatedness using GEMMA. This approach has been previously used with good results [18]. This confirmed the association of hindwing white and forewing white at chromosome 8, but given the sample size and high relatedness, the peak was broad (electronic supplementary material, figure S2 and table S2).

Following that, we set out to narrow down the associated region in *H. bolina* and identify the locus controlling the presence of white elements in *H. misippus*. We analysed whole-genome data from 335 *H*. *misippus* and 214 *H*. *bolina* individuals varying in hindwing and forewing coloration from Orteu *et al*. [33] and performed a GWAS for variation in hindwing white, using separate reference genomes for each species (electronic supplementary material, tables S3 and S4). The highest peak of association for hindwing white (and for forewing white in *H. bolina*) was on chromosome 8 in both *H. misippus* and *H. bolina* (figure 1*b,c*). As chromosomes in both genomes were named based on homology to the *M. cinxia* genome, these are homologous chromosomes, but coordinates within those chromosomes are not comparable between the two reference genomes. To explore possible candidate genes for the control of the trait and clarify homology between the two associated regions, we surveyed the genes annotated near each of the associated regions, revealing that *cortex/ivory/mir-193* were the clear candidates in both cases (figure 1*d,e* and electronic supplementary material, tables S5 and S6). SNPs associated with hindwing polymorphism in both species fall in the non-coding sequence around *cortex/ivory/mir-193*. On the other hand, the region associated with forewing phenotype in *H. bolina* is broad and covers the upstream and downstream regions and *cortex* itself, making it impossible to determine where the causal mutation is.

In *H. missippus*, there is a second peak of association seen in the Z chromosome. This could be a second major effect allele contributing to variation in the trait, which would fit the previous hypothesis of two loci, the S and A loci, controlling hindwing colour variation in *H. misippus*. The top six SNPs fall in the intron of a gene (g10224), which could be linked to this phenotype but is uncharacterized in *Heliconius melpomene* and *Drosophila melanogaster* (similarity assessed by BLASTp; gene IDs HMEL010373g1 and NP_572291). A gene in the vicinity, g10223, is identified as a cytochrome b-c1 complex subunit Rieske (*HMEL010374g1* in *H. melpomene*), which could be a better candidate. However, further work is necessary to explore the association peak and its candidate genes.

In contrast to *H. bolina*, where the same genomic region controls both forewing and hindwing white, variation in forewing phenotype in *H. misippus* is controlled by an unlinked major effect Mendelian locus, the M locus on chromosome [33]. The dominant *M* allele produces the black-and-white *misippus* wings, while recessive *mm* homozygotes can have either the all-orange *inaria* wings or intermediate *immima* wings. The switch between *inaria* and *immima* wings has been hypothesized to be controlled by the A locus (i.e. an epistatic interaction with the M locus, in addition to its effect on hindwing white). To identify the locus controlling the switch between *inaria* and *immima* morphs, we performed a GWAS but found no genomic regions associated with the two morphs (electronic supplementary material, figure S4). Furthermore, to test specifically whether the hindwing A locus is associated with these forewing phenotypes, we analysed the relationship between haplotypes at the A locus region described above. Using neighbour-joining trees of varying window sizes, we found no structure among the haplotypes distinguishing the *inaria* and *immima* morphs. This suggests that the hypothesis of an epistatic effect of the A locus on forewing phenotype may be incorrect. However, our genealogical analysis should be interpreted with caution as it is based on haplotypes inferred through phasing and imputation of low coverage (average of 1×) linked-read sequencing data.

### (c) No large rearrangements are present at the associated locus in *H. misippus*

In some cases in which *cortex/ivory/mir-193* has been associated with wing phenotype variation, chromosomal rearrangements such as inversions have been shown to be present. For example, multiple inversions around (and including) *cortex* are involved in morph diversity in *Heliconius numata* and in oakleaf butterflies of the *Kallima* genus [19,55]. We examined whether there was any structural variation associated with hindwing white using Wrath, a program for the analysis of linked-read data [33]. We analysed barcode sharing between genomic windows and generated heatmaps to explore the presence of rearrangements in the dataset. First, we used a small window size (100 bp) to explore the region along and around the associated locus and did not detect any signs of structural variants present in the data (electronic supplementary material, figure S5). Using a small window size allows for a fine-grained detection of rearrangements, but computational requirements limit the application to only small genomic regions. Then, we analysed the whole of chromosome 8 with a larger window size, with which we would be able to detect larger rearrangements in the dataset, and did not detect any rearrangements near *cortex/ivory/mir-193* (electronic supplementary material, figure S5). The number of samples used for each phenotype, 104 individuals with white hindwings and 228 with orange ones, should be enough to detect any rearrangements present at the associated locus, as has been shown for the M locus in Orteu *et al*. [33]. However, an insertion in the non-reference allele cannot be ruled out, as rearrangements can only be detected through the mapping signatures present in the reference genome.

### (d) The elements controlling hindwing white coloration in *H. bolina* and *H. misippus* are not homologous

**Figure 2.**
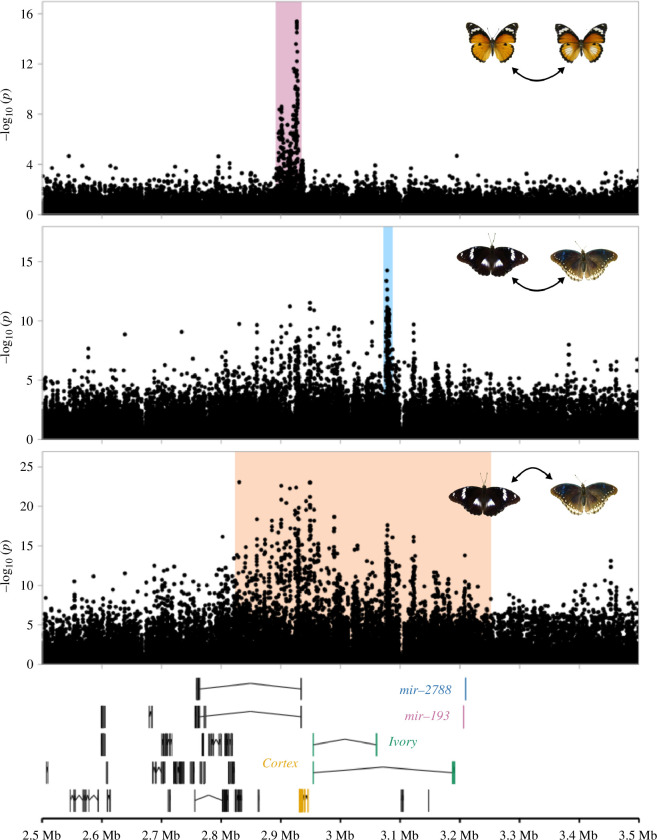
Non-coding regions associated with hindwing white are not homologous between *H. bolina* and *H. misippus*. Mapping of *H. misippus* and *H. bolina* whole-genome data to the *H. misippus* reference genome (*HypMisi_v2*) and generating a GWAS for hindwing and forewing white in the two species show that the loci associated with hindwing white in the two species are not overlapping, while the locus associated with forewing white in *H. bolina* is broad and overlaps the locus associated with hindwing white in *H. misippus*.

Next, we explored the evolutionary history of hindwing colour in the two species. More specifically, we wanted to test whether haplotypes associated with hindwing coloration in *H. bolina* and *H. misippus* were homologous. We first defined regions around the top associated SNPs in each species for hindwing and forewing white (figure 1*d,e*). In *H. bolina*, we defined a region of 343 476 bp (chromosome 8: 14 149 517–14 492 993) for the forewing white association and a region of 6149 bp (chromosome 8:14 145 391–14 151 540) for hindwing white, while for *H. misippus* the region defined was of 33 000 bp (chromosome 8:2 897 000–2 930 000) in the hindwing white GWAS. Then, to infer sequence homology, we aligned the two reference genomes using Satsuma2 and looked for alignment tracts overlapping the defined regions of association (figure 1*d,e*, lines between plots). The regions of the highest association with hindwing white did not seem to be homologous based on the alignment. That is, the region containing the top associated SNPs in *H. bolina* maps to a region of chromosome 8–125 kbp away from the region of top associated SNPs in *H. misippus*. Contrastingly, the region of highest association with forewing white in *H. bolina* overlaps with the region of top associated SNPs with hindwing white in *H. misippus* (figure 1). However, whether the causal loci are homologous in the two species cannot be determined as the region associated with forewing white in *H. bolina* is too broad (approx. 343 kb; figure 1). As an additional assessment of homology, we mapped the *H. bolina* sequence reads to the *H. misippus* reference genome and repeated the GWAS (figure 2). This confirmed that the three regions of top associated SNPs are not identical in the *H. misippus* reference genome, but the association peak for *H. bolina* forewing variation is broad and overlaps the region of association in *H. misippus*. Interestingly, the associated loci for hindwing white in both species are situated at opposite sides of *cortex*: the *H. misippus* associated locus is downstream of *cortex* (3′ end), while the *H. bolina* one is upstream (5′) of *cortex*.

### (e) Two transposable element insertions at the candidate locus in *H. misippus* are associated with continuous variation in hindwing white

**Figure 3.**
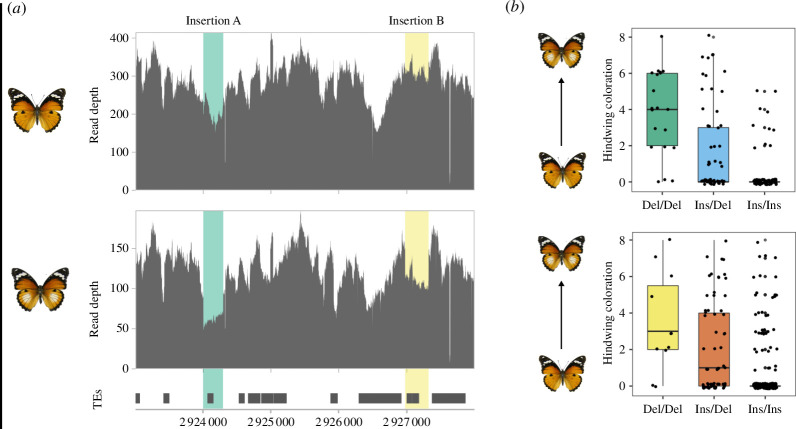
Two insertions are associated with continuous variation in hindwing white in *H. misippus*. (*a*) Read coverage at chromosome 8 shows two distinct regions of lower coverage in individuals with a hindwing white spot (middle track) compared to individuals with orange hindwings (top track). Transposable elements are found in both insertions (bottom track). Insertion A is indicated in green and insertion B in yellow. (*b*) Continuous variation in hindwing white is associated with the genotype of the insertions, where homozygous individuals for the insertion have a smaller or absent white spot in the hindwing, homozygous individuals without the insertion have larger white spots and heterozygotes are intermediates. *Y*-axis shows variation in hindwing white spot size from 8 (large) to 0 (absent) following Gordon & Smith [32].

Transposable element insertions at the non-coding region of *cortex* have been shown to be involved in wing pattern variation in *Heliconius* butterflies and in the peppered moth *Biston betularia* [16,17]. To explore whether TE insertions are involved in hindwing colour variation in *Hypolimnas*, we calculated read depth at the region of association for *H. misippus* individuals pooled by hindwing phenotype. We observed two regions with differential read depth between pooled individuals with orange hindwings and those with white spots; we named these insertion A (downstream) and insertion B (upstream; figure 3*a*). Insertion A contains an insertion of a LINE TE, while insertion B contains three consecutive TE insertions, two Helitrons and an unknown TE. This suggests that the reference genome carried the two insertions; however, its phenotype is unknown as it was sampled as a pupa [36].

Then, we determined the insertion genotype of each of the individuals by using a SNP in linkage with each insertion; we identified two SNPs, each associated with one of the insertions. Insertion A was associated with a SNP at 2 923 994 on chromosome 8, which had two alleles A (*n* = 238) and G (*n* = 130). All reads covering the SNP site and across insertion breakpoint (7:2 924 003) had the A allele (*n* = 231), while all reads presenting a G at the SNP site mapped only until the breakpoint, except for one read carrying a G and covering the first nucleotide of the insertion. The SNP at 2 927 362 on chromosome 8 presented two alleles G (*n* = 386) and T (*n* = 118). In total, 258 reads covered the SNP and the breakpoint. Of those, most of them (99.2%; *n* = 256) presented a G, while only 0.8% (*n* = 2) presented a T. When looking at the reads covering the SNP, most reads carrying a G covered the breakpoint (66.3%), while most reads carrying a T did not (98.3%).

Once we had determined the insertion genotype of the individuals, we quantified the association of the insertions with the differences in coloration in the hindwing. We observed that individuals who are homozygous for either of the insertions are more likely to not have a white spot or for it to be reduced than individuals not carrying them, while heterozygotes have intermediate phenotypes when analysing each insertion separately (ANOVA 2 d.f. X2927362 *p*‐value = 1.234 × 10^−7^ and X2923994 *p-*value = 2.016 × 10^−8^; figure 3*b*). This inheritance fits with Gordon & Smith’s [32] hypothesis for the A/S loci, as both loci were hypothesized to have incomplete dominance and variation in their penetrance. We then quantified the association of hindwing phenotype with both insertions combined and observed that individuals that are homozygous for at least one of the insertions have a reduced or absent white spot compared to those being homozygous for the absence of at least one of the insertions, while double heterozygotes presented intermediate phenotypes (ANOVA 4 d.f. *p*-value 6.185 × 10^−9^; figure 3*b*). Interestingly, out of the 128 individuals with genotypes at both diagnostic sites, none of them was homozygous for the absence of insertion at one site and homozygous for the insertion at the other.

### (f) GWAS for orange coloration in *H. bolina* points at *optix* as the main candidate

**Figure 4.**
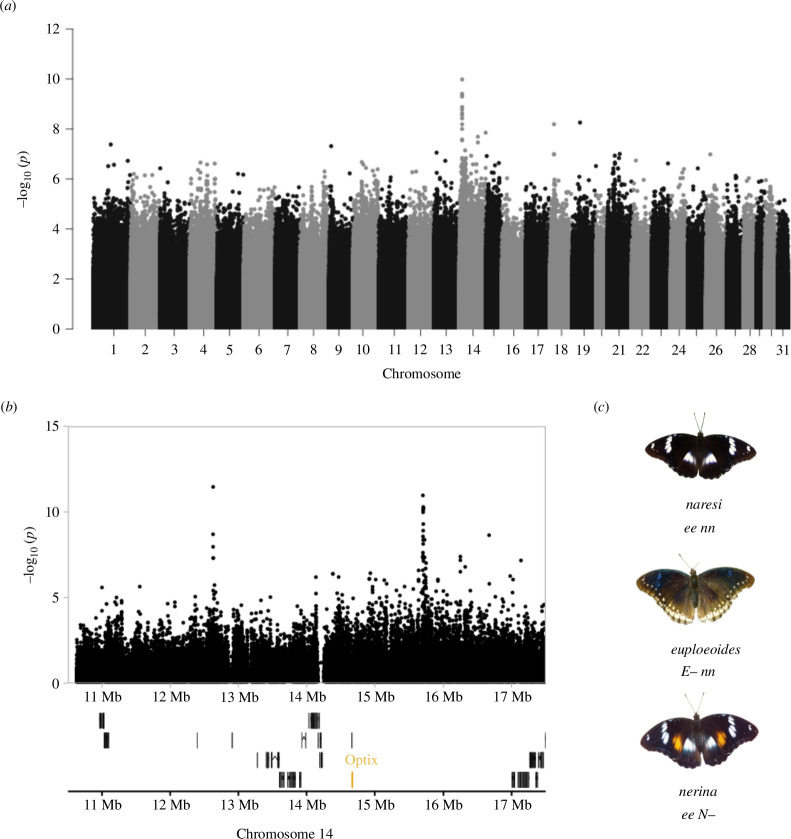
Orange forewing coloration is associated with variation near *optix* in *H. bolina*. (*a*) Genome-wide association study of orange coloration shows the highest peak of association in chromosome 14. (*b*) A zoom-in of the associated region reveals two peaks of association in non-coding loci, between which the gene *optix* is found. (*c*) The different morphs of *H. bolina: naresi*, *euploeoides* and *nerina*.

Wing pattern variation in *H. bolina* also includes variation in orange coloration in the forewing (figure 4*c*). The *nerina* morphs have an orange spot in the forewing, while the other two morphs, *euploeoides* and *naresi,* do not (figure 4*c*) [29]. This orange element is genetically determined by the N locus, whose dominant allele produces the *nerina* phenotype (figure 4*c*). Combinations between the two loci determining wing patterns in *H. bolina* generate all possible phenotypes. To identify the N locus, we performed a GWAS on the wild *H. bolina* sequenced samples. This analysis revealed a significant association peak in chromosome 14 (figure 4*a*). From the genes around the associated region, one clear candidate stood out, the gene *optix* (figure 4*b*). *Optix* has been linked to orange and red coloration in multiple species of butterflies, including some *Heliconius* species and *Vanessa cardui,* using association studies and functional testing with CRISPR, and thus is a strong candidate for the control of this phenotype [14,26].

Finally, a fourth morph exists, *pallescens*, whose phenotype might be controlled by a different allele at the N locus or by a third distinct locus [29]. However, no individuals of this phenotype were sampled, and thus no evaluation of it was possible.

## 4. Discussion

Studies of convergent evolution have revealed the repeated use of the same ‘hotspot genes’ when dissimilar species evolve similar phenotypes. Here, we have used whole-genome analysis of 645 individual butterflies to demonstrate that putative *cis*-regulatory regions near two well-known wing patterning loci, *cortex/ivory/mir-193* and *optix,* are associated with differences between Batesian mimicry morphs in *Hypolimnas* butterflies. We have found that non-coding loci around *cortex/ivory/mir-193* are associated with variation in white pattern elements in the forewing and hindwing of *H. bolina* and in *H. misippus* hindwings. The *cortex/ivory/mir-193* locus has now been implicated in controlling crypsis, warning colour and Batesian and Müllerian mimicry patterns across the Lepidoptera, making this gene a genuine ‘hotspot’ for genetic change.

*Cortex* is a cell cycle regulator that has been shown to determine scale identity, resulting in changes in pattern and coloration [16]. However, new evidence suggests that the wing patterning switch is in reality controlled by the *ivory* long non-coding RNA and the *mir-193* microRNA sitting next to *cortex* [23–25]. Here we refer to the region implicated with colour pattern as the *cortex/ivory/mir-193* locus. *Cortex/ivory/mir-193* has been repeatedly linked to changes in switches between melanic and white/yellow wing pattern elements in multiple Lepidoptera including *Heliconius* species and other butterflies and moths. For example, a single TE insertion in an intron of *cortex* has been shown to cause the switch between the peppered and melanic morphs of the peppered moth, *B. betularia* [17], while in the Batesian mimic *Papilio clytia, cortex/ivory/mir-193* has been associated with the differences between mimetic morphs: one with brown wings and reduced white elements in the apex mimicking *Euploea* models similarly to *H. bolina,* and another with melanic black and pigmented white scales in a pattern resembling toxic tiger butterflies [18]. *Cortex/ivory/mir-193* has also been linked to changes in colour phenotypes in other moths, such as the silk moth *Bombyx mori* and some geometrids, and butterflies, such as *Junonia coenia* and *Bicyclus anynana* [20,22,23,25,56,57]. Crucially, in some of those cases, *cis*-regulatory variation around *cortex* has been suggested to be the cause of the phenotypic changes*,* with two cases in which TE insertions at regulatory regions have been implicated [16,17,20]. In *Heliconius, cortex/ivory/mir-193* controls the switch between type of scales, which can be either yellow or white, and type II scales, which can be black or red [16,24]. The added effect of two other genes*, optix* and *aristaless1,* determines the final coloration of each scale type [13,14]. Thus, *cortex/ivory/mir-193* is a strong candidate for controlling the development of white pattern elements in *Hypolimnas* species.

Our study does not provide functional validation of the role of *cortex/ivory/mir-193* in *Hypolimnas*, so we cannot yet rule out the possibility that other genes around the associated locus could have a role in wing phenotype in *H. bolina* and *H. misippus*. Crucially, the genes *domeless* and *washout* found >150 kbp downstream of *cortex/ivory/mir-193* have been suggested to be involved in wing phenotype determination in *Heliconius* [16,58]. Similarly, evidence from *H. numata,* in which wing polymorphism is controlled by a supergene around *cortex/ivory/mir-193* containing three inversions, suggests that other genes around *cortex/ivory/mir-193* also have a function in wing phenotype [59].

The data in *H. bolina* suggest that there are distinct non-coding regions, and possibly *cis*-regulatory elements (CRE), controlling the presence of the forewing white band and the hindwing white spot. In reared families, we have shown that these elements can segregate independently, and they show distinct albeit partially overlapping association peaks. Strong linkage disequilibrium owing to physical linkage and potentially also recent selection could lead to longer haplotypes and a broad association peak that could contribute to this overlap. Interestingly, it seems likely that the CREs causing differences in white coloration in the hindwing are not homologous between the two species, as they map to slightly different locations. These results highlight the complexity of the region around *cortex/ivory/mir-193* and suggest that modular CREs have spatially restricted effects in *H. bolina*. These results strongly parallel those in *Heliconius*, where evidence for recombination within the locus between different elements in the same species is also found [60] and adjacent but distinct CREs have been implicated in the convergence of mimetic species [16].

Hindwing phenotype in *H. misippus* varies continuously, and wings can be completely orange or have a white spot with a highly variable size. Two major effect loci, the A and S loci, have been hypothesized to control such variation. However, using GWAS, we find evidence of only one of those loci. This could be owing to the samples present in the dataset. Furthermore, the A locus has been hypothesized to be a supergene with effects on hindwing coloration, forewing pattern (differences between *inaria* and *immima* morphs) and body size [32]. However, we find no genomic region associated with the differences between *inaria* and *immima* morphs. Nonetheless, the GWAS for hindwing white in *H. misippus* reveals a second peak of association in the Z chromosome, which could be the second locus hypothesized (A/S).

Similarly to *cortex/ivory/mir-193*, *cis*-regulatory variation around the transcription factor *optix* has been associated with colour pattern differences in *Heliconius* [14,61,62]. CRISPR–Cas9 knockouts have shown that *optix* has an effect on structural coloration and on the red and orange pattern differences in *Heliconius* species as well as in *J. coenia*, *Agraulis vanillae* and *V. cardui* [26]. This highlights the widespread importance of *cortex* and *optix* in the evolution of wing patterns across diverse Lepidoptera species.

Goldschmidt [63] proposed that mimicry could be favoured by shared developmental systems, in which single mutations could activate ancestral developmental pathways to create the same phenotype in the model and mimic. In *Heliconius,* it has been shown that convergent mimicry does indeed result from allelic variation at the same few hotspot loci [14–16]. However, as the convergence is between species in the same genus, perhaps it is not surprising that these species show a similar developmental basis. *Hypolimnas* offers an opportunity to study Batesian mimicry between far more distantly related species. Unlike in Müllerian systems, Batesian mimicry has a clear model and mimic, so there is a clear hypothesis for the order in which evolutionary divergence has occurred. The fact that *H. misippus* mimics the four morphs of *D. chrysippus* makes it an ideal system to investigate if convergence in phenotype results from similar molecular changes in more distantly related species (84 Myr [64]).

Crucially and similarly to Goldschmidt [63], Bernardi [65] and Pierre [66] suggested that mimetic patterns of female *H. misippus* are ancestral and the male pattern derived, and propose the unlikely hypothesis that the female phenotypes of *H. misippus* are homologous to those of *D. chrysippus,* dating back to a time of common ancestry. The identification of the loci controlling wing phenotypes in both species is crucial to shed light to these hypotheses. In *D. chrysippus,* wing phenotype is controlled by three main loci A, B and C [67,68]. While the B and C loci control forewing phenotype and are part of a supergene found in chromosome 15, the A locus controlling hindwing variation is found in chromosome 4 [69]. Crucially, none of these loci is close to *cortex* (chromosome 8) or *optix* (chromosome 14). This contrasts with our results showing that hindwing variation in white coloration is likely controlled by *cortex* at chromosome 8 and possibly by a locus at the Z chromosome and that forewing mimicry is associated with a locus at chromosome 29 [33]. Overall, these results indicate that the convergence in wing phenotype seen in *H. misippus* to mimic *D. chrysippus* does not have a homologous genetic basis and opposes the ideas of Goldschmidt [63], Bernardi [65] and Pierre [66].

Black wings with white elements akin to those seen in the *naresi* morph of *H. bolina* and *H. misippus* and *H. bolina* males are common among *Hypolimnas* butterflies [33]. Given that the same region of chromosome 8 seems to be controlling the presence of white pattern elements in *H. bolina* and *H. misippus* at least in the hindwing, it could be that this control is ancestral in the genus, with *cortex/ivory/mir-193* having a common function. However, *H. bolina’s* hindwing and forewing white patterning loci are not homologous, thus suggesting independent evolution of the two. Furthermore, while hindwing white coloration has a clear mimetic benefit in *H. misippus*, that is not the case in *H. bolina*, in which the only morph, *euploeoides*, does not present hindwing white coloration or a subapical forewing band. This could suggest that the mimetic morph is derived in *H. bolina* and that the reduction of hindwing white could have evolved independently in the two species.

Taken together, our results highlight the importance of ‘genetic hotspots’ in the evolution wing phenotypes and particularly of *cortex/ivory/mir-193* and *optix* in determining wing patterns in diverse Lepidoptera species. More generally, they add to the evidence showing that convergent phenotypes are often the result of repeated evolution at the genetic level (i.e. genetic parallelism or convergence) [1,3]. Exploring the genetic basis of other adaptive phenotypes in diverse clades is necessary to clarify if this genetic re-use is as generalized as the current evidence suggests.

## Data Availability

Whole-genome sequencing samples of *Hypolimnas misippus*, *Hypolimnas bolina* and other *Hypolimnas* are available at NCBI BioProject accession PRJEB64669. Supplementary material is available online [70].
